# An Editor's rave review

**DOI:** 10.1101/gad.350468.123

**Published:** 2023-01-01

**Authors:** Stephen J. Elledge

**Affiliations:** Division of Genetics, Department of Medicine, Howard Hughes Medical Institute, Brigham and Women's Hospital, Department of Genetics, Harvard Medical School, Boston, Massachusetts 02115, USA

I am delighted to participate in this celebration of Terri Grodzicker and her role as the Editor of *Genes & Development*. Soon after I began publishing in *G&D*, I came to admire her as a skilled Editor and a keen observer of the human condition. Beyond her role as Editor of *G&D*, Terri played many other important roles at Cold Spring Harbor Laboratory: as a lab head, as an organizer of the many meetings at CSHL with David Stewart, and currently as the Dean of Academic Affairs, among others. In order to understand *G&D*, it is important to understand Terri's scientific history because it shaped her view of science, and that, in turn, shaped *G&D*. Terri obtained her PhD during the golden era of bacterial genetics, at which time she worked on polarity in the lac operon at Columbia University. She went on to a postdoctoral position with Jon Beckwith at Harvard Medical School, where she studied the roles of adenylate cyclase and the CAP protein in both bacteriophage λ latency and lac operon repression. She then went on to CSHL, where she made the leap into mammalian biology by studying eukaryotic viral genetics first with Joe Sambrook and then as a Staff Scientist at CSHL, where she led a group studying adenovirus and SV40 genetics and their roles in cancer. This background in the rigors of bacterial genetics and eukaryotic systems provided her with a wide breadth of experiences. That—in addition to being embedded into the scientifically rich environment of CSHL, steeped in the history of molecular biology—put her in the perfect position to oversee one of the most prestigious journals in the biological sciences.

Of course, I knew nothing about all that when I first came in contact with Terri indirectly through my first submissions to *G&D* in 1989 and 1990. I need to describe that because it taught me several important lessons that made a lasting impact on my scientific career. I call it “The story of the accidental great paper.”

I was a postdoc in Ron Davis’ lab at Stanford University, where I worked on many topics—especially technology development, which I loved. The problem I wished to solve was how to identify and clone eukaryotic sequence-specific DNA binding proteins—a pressing need at the time. I had developed a bacterial reporter in which I used transcription collision to turn off the expression of a drug resistance marker using an opposing strong promoter, a novel finding. Repressing that strong promoter resulted in activation of the aadA gene to give spectinomycin resistance and thus a selection for the expression of a sequence-specific DNA binding protein, which I showed worked with several bacterial repressors and the eukaryotic EBNA1 protein. However, in order to make it work, I first had to figure out where I could place the DNA binding site for efficient repression. So, I systematically moved the Lac or Trp operators different distances from the start of transcription and examined repression. I found that there were distinct plateaus of repression and that they corresponded to known in vitro steps in RNA polymerase initiation at a promoter that also seemed like a publishable unit. Coincidentally, I had just read a *Cell* paper from the Ptashne lab about splicing eukaryotic transcriptional activation domains to a bacterial repressor that seemed to be written way over the top, touting its own significance. Just for fun, I thought I would write my repressor positioning paper that way, even tracing the significance of the problem back to Jacob and Monod. I went out of my way to wring out every drop of significance while pointing out that it was in vivo evidence for the steps in RNA polymerase activation previously seen only in vitro. I cosubmitted my papers, the exciting new method, and basically the control experiments for the method. Much to my surprise, *G&D* rejected the new method paper but accepted the second paper and, in fact, commented on how well it was written. I learned three things from that experience. First, Terri was not swayed by shiny technical objects—an observation driven home in subsequent failed submissions. Second, it makes a very big difference if you set up the problem you are addressing in its historical context and make the effort to explain exactly what you have shown beyond what was known and why it is important. Third, Terri and *G&D* had an eye for serious science that is written to communicate its significance clearly. A very happy accident and something very important to learn early on. Oh, and a tip of the hat to Brent and Ptashne.

Science Editors come in many flavors. Some are seasoned and efficient. Others are quite inexperienced, having recently left academia, and are now tasked with overseeing research outside of their areas of expertise. This can result in an overreliance on the reviewers, many of whom are senior to them and do have expertise in these areas. This sets up a problematic situation in which the reviewers run the show. The inexperienced Editor does not wish to alienate the reviewers and is also fearful of making a mistake in accepting a paper that could have a problem or lacked enough pizzazz for the boutique journals. Instead of diving deep, learning the key science, and evaluating the reviewers’ comments, they shuffle off the reviews to authors, often with no guidance other than a boilerplate letter explaining that the paper is not acceptable in its current form, etc., and the implicit demand that they address every single item requested by the reviewers—or else. It is the easiest way to protect themselves from criticism from above and does not require the time-intensive effort of reading the paper and deciding what needs to be done. This is compounded by the fact that many Editors are overworked and have too many papers to handle at once (an issue of the business model I suspect), and this leads to a high burnout rate. This is a lose–lose situation: Either the author loses by having the paper rejected, sometimes unjustly, or they lose by spending inordinate amounts of time and money doing experiments that do not change the central message of the paper. As a result, some papers have submission and acceptance dates separated by over a year. I recently had a paper that took over a year for which they asked us to submit a revised manuscript three times, each of which went back to one or more reviewers, and the take-home message was the same as the initial submission. The Editor could not be bothered to make an editorial decision and say enough is enough. While there are now efforts afoot to create open access publication models, that alone does not solve some of these issues. Some Editors gain the confidence to make independent decisions; others never do.

Terri, on the other hand, was the epitome of a great Editor. She knew good science when she saw it and was reasonable about what needed to be done and what didn't, often overruling reviewers. She could do that because she read the papers and knew the science, having a deep background of experience in different fields. She had a sense for when something was good and acted on it using trusted reviewers. In that respect, she shared that ability with her then major competitor at *Cell*, Ben Lewin. A major and refreshing difference between the two was that Terri did not play games and manipulate the authors. Editors hold a lot of power over scientists whose careers can be significantly impacted. This is especially true of Editors at top journals who can, in essence, indirectly choose who gets hired by certain departments and sometimes even who gets tenure. This is a sorry state of affairs but is due in no small measure to the academic departments and faculties themselves, who defer far too easily to the journal name relative to the science itself. There are also broader psychological issues at play that are too complex to go into detail here. Suffice it to say that Terri Grodzicker was a fair and levelheaded Editor. She would accept a paper in principle if you addressed certain issues that were germane to the story's conclusions. She made the decision. Authors were not enslaved to reviewers. This made publishing an enjoyable experience.

After sending some of my best work to *G&D*, I began to review for them. Reviewing papers is an essential component of the scientific publishing ecosystem in which the reviewer and Editor enter into a symbiotic relationship from which both can benefit. As a young faculty member, I felt honored to be asked to review. Perhaps I was a tad naïve as to exactly who was doing a favor for whom (see below). Nevertheless, I reviewed a lot of papers and turned them around quickly. One day, I reviewed a paper from a prominent cancer researcher at MIT that had merit but lacked nearly every proper control for each experiment. A few weeks later, Terri said the senior author had complimented my review as being particularly spot on. Perhaps he recognized the fingerprints of MIT's Method and Logic course on how to read the literature, a course he sometimes taught. Terri then asked me to join the Editorial Board. As a board member, I thought I should review every paper when requested, and Terri asked frequently. A few years later, I got a package in the mail with a gift for winning the award for reviewing the most papers for *G&D* that year. After that, I stopped reviewing so many papers. The take-home message here: If you are young and want to become an Editorial Board member, review thoroughly, fairly, and quickly, and Editors will begin to trust and rely on your opinions. In addition, recognize your place in the scientific publishing ecosystem and don't overdo it. Ecosystems need balance, and so do you.

One of the most distressing aspects of publishing from an author's point of view is getting scooped. It is a policy at many journals that if a competing paper comes out while your paper is in review, they can and will reject your paper. If the author is connected and gets wind of a competing paper, they might be able to speed up their paper, as that is a game some Editors play, but it only works for the well-heeled labs. I have ended up on both sides of this truly toxic policy—the scooper and the scoopee. Both outcomes leave a bad taste in one's mouth, and it brings out the worst competitive urges in scientists. Scientists want to be first, but it should never be a “winner take all” situation. Terri solved that problem for *G&D* by adopting a policy of not rejecting a paper in review because a related competing paper came out. I don't know whether Terri simply decided to do the right thing based on her experiences as a scientist and author or it was pure business acumen realizing that offering something other journals were not would bring more authors with competitive papers to *G&D*. Regardless, it made a world of difference in the experience of publishing at *G&D*. I wish every journal would follow her lead.

My colleague Titia de Lange once remarked that when she retired, she wanted to start a website entitled “ratemyeditor.org.” That is great idea. I would like to submit the first review. For Terri Grozicker, I give five stars! Thanks for so many great years as a shining light leading us through the darkness of scientific publishing.

